# Supplementary material for: Complex‐centric proteome profiling by SEC‐SWATH‐MS
Source: Mol Syst Biol. 2019 Jan 14;15(1):e8438. doi: 10.15252/msb.20188438 (PMC6346213; doi:10.15252/msb.20188438)
Supplement: Supplementary file 7 — Dataset EV6 [file MSB-15-e8438-s007.zip › feature_plots_bioplex/P62241.pdf]

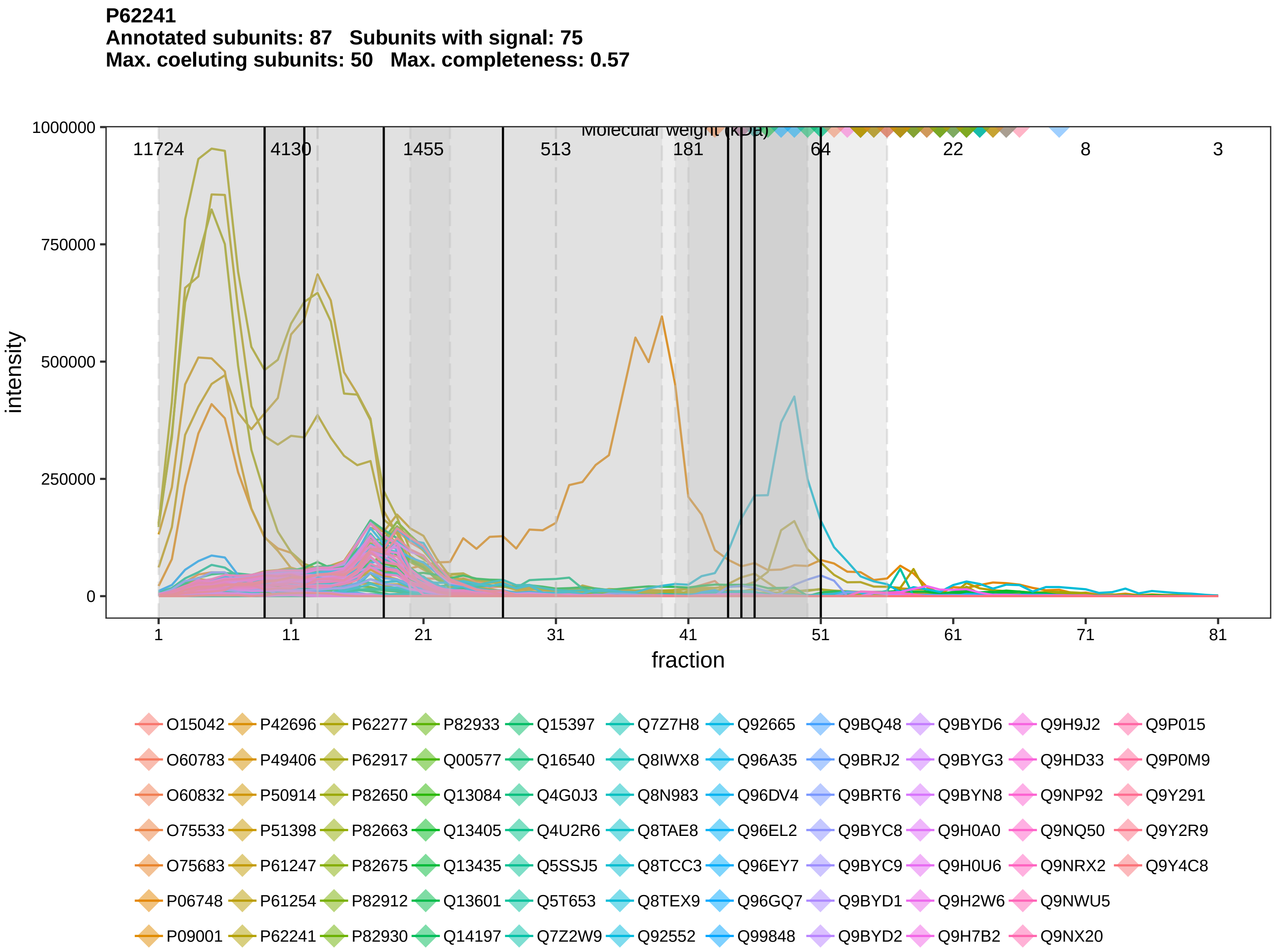
P62241
Annotated subunits: 87 Subunits with signal: 75
Max. coeluting subunits: 50 Max. completeness: 0.57
Molecular weight (kDa)
11724
4130
1455
513
181
64
22
8
3
intensity
1000000
750000
500000
250000
0
fraction
1
11
21
31
41
51
61
71
81
O15042
O60783
O60832
O75533
O75683
P06748
P09001
P42696
P49406
P50914
P51398
P61247
P61254
P62241
P62277
P62917
P82650
P82663
P82675
P82912
P82930
P82933
Q00577
Q13084
Q13405
Q13435
Q13601
Q14197
Q15397
Q16540
Q4G0J3
Q4U2R6
Q5SSJ5
Q5T653
Q7Z2W9
Q7Z7H8
Q8IWX8
Q8N983
Q8TAE8
Q8TCC3
Q8TEX9
Q92552
Q92665
Q96A35
Q96DV4
Q96EL2
Q96EY7
Q96GQ7
Q99848
Q9BQ48
Q9BRJ2
Q9BRT6
Q9BYC8
Q9BYC9
Q9BYD1
Q9BYD2
Q9BYD6
Q9BYG3
Q9BYN8
Q9H0A0
Q9H0U6
Q9H2W6
Q9H7B2
Q9H9J2
Q9HD33
Q9NP92
Q9NQ50
Q9NRX2
Q9NWU5
Q9NX20
Q9P015
Q9P0M9
Q9Y291
Q9Y2R9
Q9Y4C8
